# Supplementary material for: A single mutation in dairy cow-associated H5N1 viruses increases receptor binding breadth
Source: Nat Commun. 2024 Dec 30;15:10768. doi: 10.1038/s41467-024-54934-3 (PMC11685663; doi:10.1038/s41467-024-54934-3)
Supplement: Supplementary file 1 — Supplementary Information [file 41467_2024_54934_MOESM1_ESM.pdf]

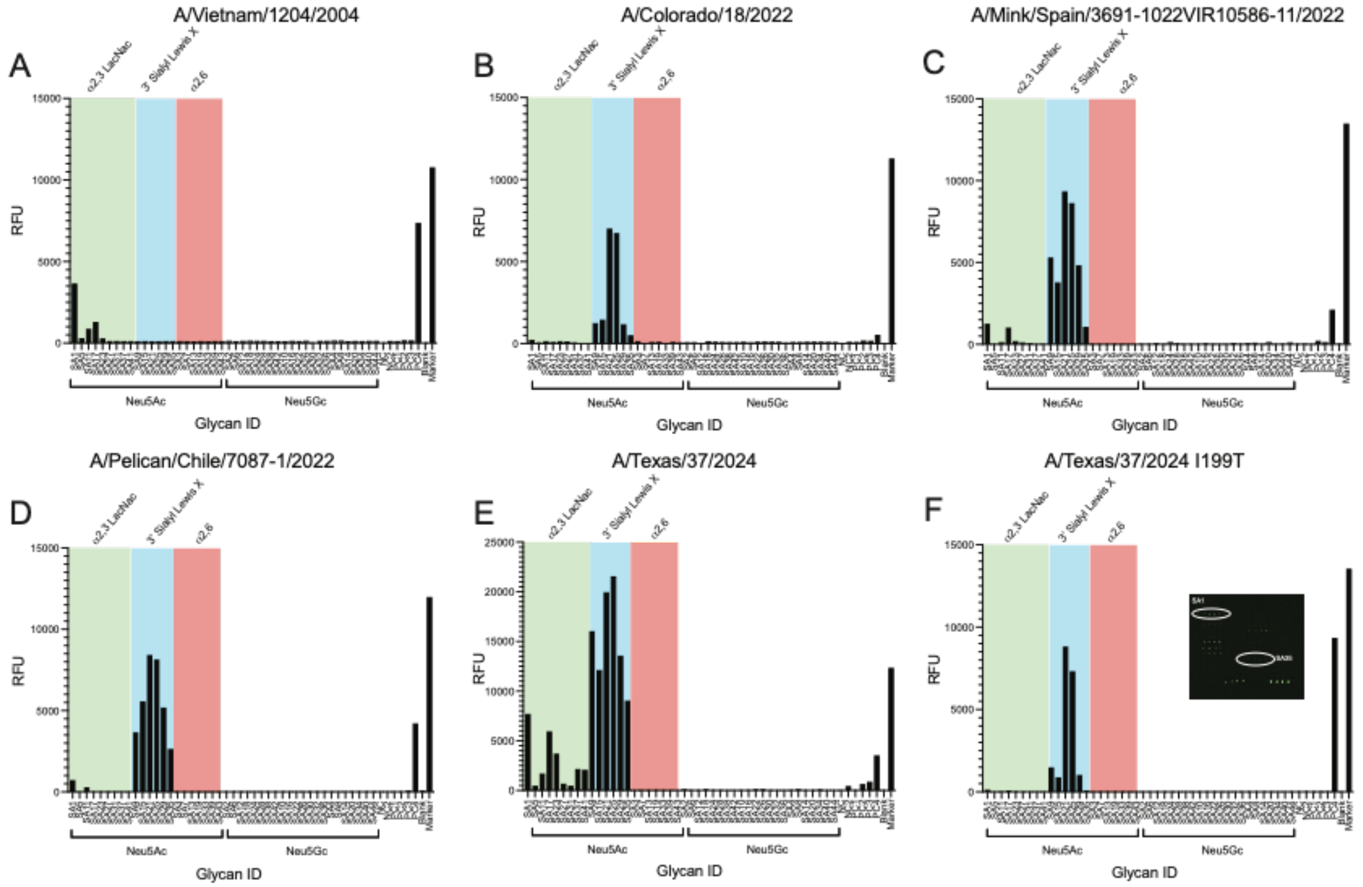

Supp. Figure 1: rHA binding to glycan microarray. (A-F) Average RFUs for H5 binding each glycan on the microarray. On the x-axis, glycans are grouped by Neu5Ac and Neu5Gc classification, and then by terminal sialic acid; a2,3 in cool colors and a2,6 in red. Additionally, a2,3 glycans have been split into a2,3 LacNac glycans (green) and 3' Sialyl Lewis X (aqua). rH5s tested are A/Vietnam/1204/2024 (A), A/Colorado/18/2022 (B), A/Mink/Spain/3691-1022VIR10586-11/2022 (C), A/Pelican/Chile/7087-1/2022 (D), A/Texas/37/2024 (E), and A/Texas/37/2024 I199T (F). Microarray fluorescence is shown in F, with SA1 and SA35 circled to indicate detection of fluorescence despite low RFU values. Array controls are as follows: Negative Controls (NC) 1 and NC2 are immobilization buffer 1 and 2; PC1-4: PC1 is biotinylated PEG, PC2 is Human IgG, PC3 is Mouse IgG, PC4 is Rabbit IgG; Blank is no sample or native surface only; Marker is streptavidin, Cy3 and Cy5 conjugates. PC4 functions as the positive control well for detection of the secondary antibody.

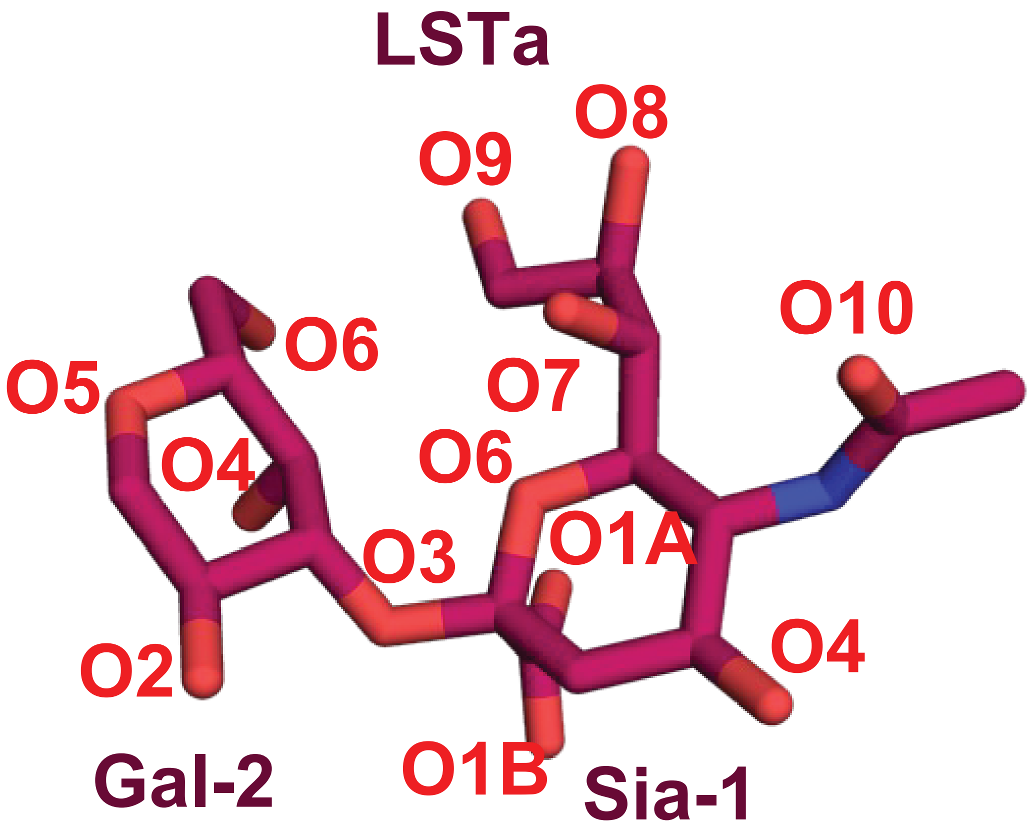

Supp. Figure 2: Annotation of LSTa.

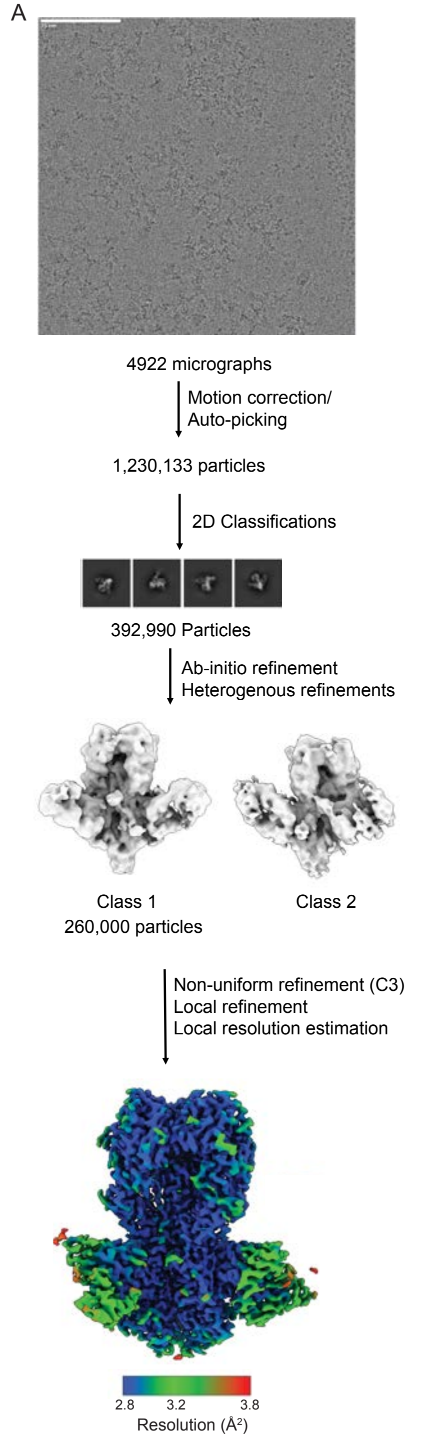

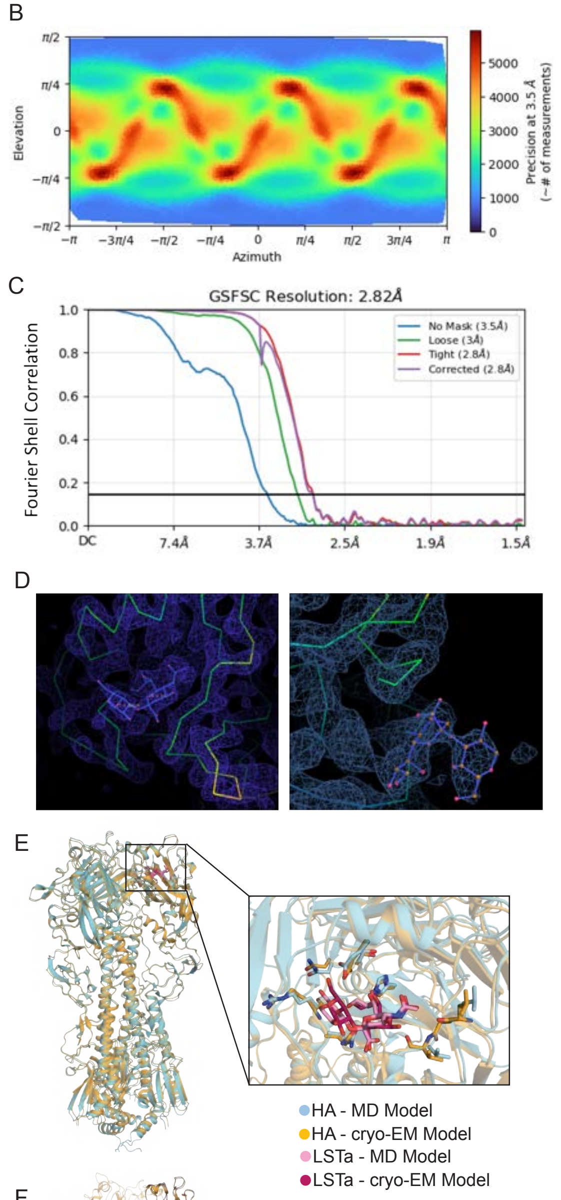

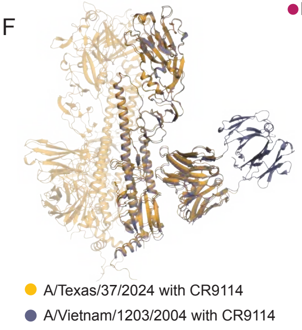

CR9114 Residues		H5 Residues A/Texas/37/2024
HCDR1	T28	N53 (HA2)
	N31	Q42 (HA2)
	N31	D46 (HA2)
HCDR2	F54	H38 (HA1)
	F54	W21 (HA2)
HCDR3	Y98	D19 (HA2)
	Y98	Q42 (HA2)
	Y99	D19 (HA2)
	Y100A	D19 (HA2)
HFR3	D72	S291 (HA1)

Supp. Figure 3: Additional data on cryo-EM structure of rH5 from A/Texas/37/2024. (A) Cryo-EM data processing workflow with representative micrograph, reference free 2D classification followed by Ab-initio and heterogenous refinements (representative classes shown), Non-Uniform (NU) refinement on the best class with C3 symmetry. The final map is colored based on the local resolution estimation. (B) Angular distribution plot from the final NU refine job. (C) Fourier Shell Correlation of rH5 in complex with CR9114 and LSTa. (D) Electron density map of the rH5 RBS and LSTa. (E) Overlay of the most probable representative MD simulation and cryo-EM structure

of rH5 binding to LSTa. (F) Overlay of CR9114 binding to A/Texas/37/2024 and Vietnam/1203/2004 (PDB: 4FQI) with CR9114 contacts with rH5 from A/Texas/37/2024.

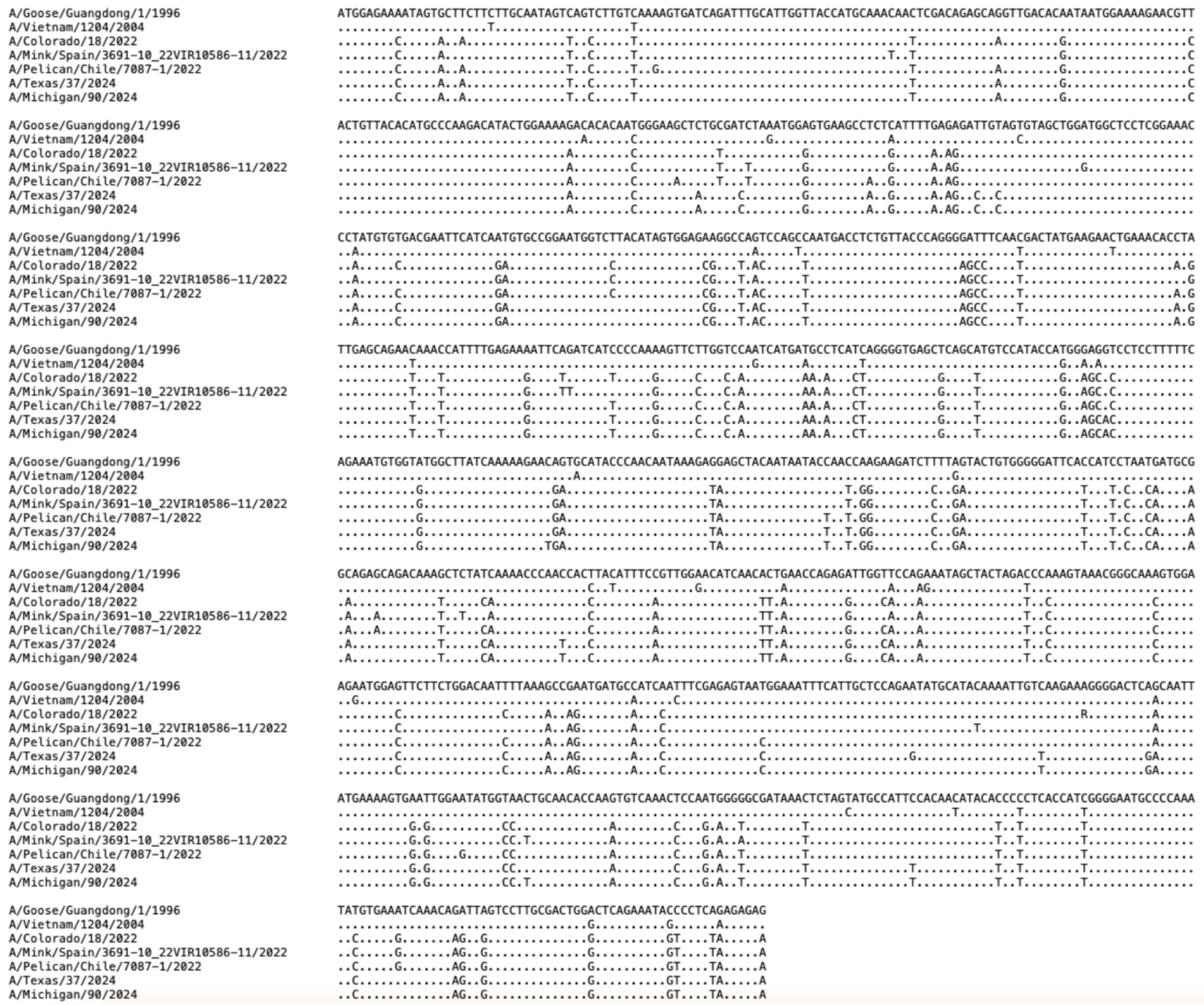

Supp. Fig. 4: Nucleotide alignments of HA1 of HAs shown in Fig. 4C.

Supp. Table 1. CryoEM data collection, processing and model building statistics.

**Map**	**HA/Texas/37/2024 + CR9114 and LSTa**
EMDB	EMD-47241
**Data collection**	
Microscope	TFS Glacios
Nominal magnification	190,000x
Voltage (kV)	200
Total dose (e^-/Å^2)	41.63
Defocus range (μm)	-0.8 to -1.6
Detector	TFS Falcon 4
Recording mode	Counting
Movie micrograph pixelsize (Å)	0.725
Number of frames (Falcon 4 EER fractions)	40
**EM data processing**	
Number of movie micrographs	4922
Number of molecular projection images in map	260000
Symmetry	C3
Map pixel size	0.725
Map resolution (FSC 0.143; Å)	2.85
Map sharpening B-factor (Å^2)	-50
**Structure building and validation**	
Model composition	
Non-hydrogen atoms	17562
Protein Residues	2211
Ligands	NAG:15<br>GAL:3<br>SIA:3
MolProbity score	1.32
Clashscore	3.72
Map correlation coefficient	0.76
EMRinger score	4.27
d FSC model (0.5; Å)	3.04
RMSD from ideal	
Bond length (Å)	0.006
Bond angles (˚)	1.100
Ramachandran plot	
Favored (%)	97.12
Allowed (%)	2.88
Outliers (%)	0.00
Side chain rotamer outliers (%)	0.00
Cβ outliers (%)	0.00
PDB ID	9DWE
