# Supplementary material for: A single mutation in dairy cow-associated H5N1 viruses increases receptor binding breadth
Source: Nat Commun. 2024 Dec 30;15:10768. doi: 10.1038/s41467-024-54934-3 (PMC11685663; doi:10.1038/s41467-024-54934-3)
Supplement: Supplementary file 2 — Description of Additional Supplementary Files [file 41467_2024_54934_MOESM2_ESM.docx]

File Name: Supplementary Data 1

Description: The accession numbers and strains used to generate the phylogenetic tree analysis in Figure 1.

File Name: Supplementary Data 2

Description: Glycans in the Neu5Ac and Neu5Gc microarray.

File Name: Supplementary Data 3

Description: MD molecular coordinates for A/Colorado/18/2022 H5, A/Texas/37/2024 H5, and A/Texas/37/2024 I199T H5.
